# Lipid Metabolism Links Nutrient-Exercise Timing to Insulin Sensitivity in Men Classified as Overweight or Obese

**DOI:** 10.1210/clinem/dgz104

**Published:** 2019-10-19

**Authors:** Robert M Edinburgh, Helen E Bradley, Nurul-Fadhilah Abdullah, Scott L Robinson, Oliver J Chrzanowski-Smith, Jean-Philippe Walhin, Sophie Joanisse, Konstantinos N Manolopoulos, Andrew Philp, Aaron Hengist, Adrian Chabowski, Frances M Brodsky, Francoise Koumanov, James A Betts, Dylan Thompson, Gareth A Wallis, Javier T Gonzalez

**Affiliations:** 1 Department for Health, University of Bath, Bath, United Kingdom; 2 School of Sport, Exercise and Rehabilitation Sciences, University of Birmingham, Birmingham, United Kingdom; 3 Department of Health Sciences, Faculty of Sport Sciences and Coaching, Universiti Pendidikan Sultan Idris, Perak, Malaysia; 4 Institute of Metabolism and Systems Research, University of Birmingham, Birmingham, United Kingdom; 5 Diabetes & Metabolism Division, Garvan Institute of Medical Research, Sydney, New South Wales, Australia; 6 Department of Physiology, Medical University of Bialystok, Bialystok, Poland; 7 Division of Biosciences, University College London, London, United Kingdom

## Abstract

**Context:**

Pre-exercise nutrient availability alters acute metabolic responses to exercise, which could modulate training responsiveness.

**Objective:**

To assess acute and chronic effects of exercise performed before versu*s* after nutrient ingestion on whole-body and intramuscular lipid utilization and postprandial glucose metabolism.

**Design:**

(1) Acute, randomized, crossover design (Acute Study); (2) 6-week, randomized, controlled design (Training Study).

**Setting:**

General community.

**Participants:**

Men with overweight/obesity (mean ± standard deviation, body mass index: 30.2 ± 3.5 kg⋅m^-2^ for Acute Study, 30.9 ± 4.5 kg⋅m^-2^ for Training Study).

**Interventions:**

Moderate-intensity cycling performed before versus after mixed-macronutrient breakfast (Acute Study) or carbohydrate (Training Study) ingestion.

**Results:**

Acute Study—exercise before versus after breakfast consumption increased net intramuscular lipid utilization in type I (net change: –3.44 ± 2.63% versus 1.44 ± 4.18% area lipid staining, *P* < 0.01) and type II fibers (–1.89 ± 2.48% versus 1.83 ± 1.92% area lipid staining, *P* < 0.05). Training Study—postprandial glycemia was not differentially affected by 6 weeks of exercise training performed before versus after carbohydrate intake (*P* > 0.05). However, postprandial insulinemia was reduced with exercise training performed before but not after carbohydrate ingestion (*P* = 0.03). This resulted in increased oral glucose insulin sensitivity (25 ± 38 vs –21 ± 32 mL⋅min^-1^⋅m^-2^; *P* = 0.01), associated with increased lipid utilization during exercise (*r* = 0.50, *P* = 0.02). Regular exercise before nutrient provision also augmented remodeling of skeletal muscle phospholipids and protein content of the glucose transport protein GLUT4 (*P* < 0.05).

**Conclusions:**

Experiments investigating exercise training and metabolic health should consider nutrient-exercise timing, and exercise performed before versus after nutrient intake (ie, in the fasted state) may exert beneficial effects on lipid utilization and reduce postprandial insulinemia.

Postprandial hyperinsulinemia and associated peripheral insulin resistance are key drivers of metabolic diseases such as type 2 diabetes (T2D) and cardiovascular disease (1–3). Obesity and a sedentary lifestyle are independently associated with changes in skeletal muscle that can reduce insulin sensitivity (4, 5) and increase hyperinsulinemia, contributing to elevated cardiovascular disease risk (2). Therefore, increasing insulin sensitivity and reducing postprandial insulinemia are important targets for interventions to reduce the risk of metabolic disease.

Regular exercise training represents a potent strategy to increase peripheral insulin sensitivity and reduce postprandial insulinemia (6). The beneficial effects of exercise on oral glucose tolerance and insulin sensitivity can be attributed to both an acute phase (during and straight after each bout of exercise performed) and the more enduring molecular adaptations that accrue in response to regular exercise (7). A single bout of endurance-type exercise activates contractile pathways in exercising muscle, which (independently of insulin) translocate the glucose transporter, GLUT4, to the plasma membrane and transverse tubules to facilitate increased transmembrane glucose transport (8–10). The mechanisms that underlie the exercise-training–induced increases in oral glucose insulin sensitivity (OGIS) include an increase in the total amount of time spent in the acute phase (7) and they also include other adaptations such as changes in body composition (eg, increased fat-free mass and reduced adiposity), an increased mitochondrial oxidative capacity (11), adaptations relating to glucose transport and insulin signaling pathways (12), and alterations to the lipid composition of skeletal muscle (13, 14).

Despite the potential for exercise to increase whole-body and peripheral insulin sensitivity, there can be substantial variability in the insulin-sensitizing effects of fully supervised exercise training programs (15). Crucially, this interindividual variability for postprandial insulinemia following exercise training has also been shown to be greater than that of a (no-exercise) control group (15), which demonstrates that some of this variability to exercise is true interindividual variability (16). Nutritional status and thus the availability of metabolic substrates alter metabolism during and after exercise (17–20). Specifically, carbohydrate feeding before and during exercise can potently suppress whole-body and skeletal muscle lipid utilization (18, 21) and blunt the skeletal muscle messenger RNA (mRNA) expression of several genes involved for many hours postexercise (22–24). This raises the possibility that nutrient-exercise interactions may regulate adaptive responses to exercise training and thus contribute to the apparent individual variability in exercise responsiveness via skeletal muscle adaptation and/or pathways relating to substrate metabolism.

Emerging data in lean, healthy men suggest that nutrient provision affects adaptive responses to exercise training (25, 26). However, feeding and fasting may exert different physiological responses in people who are overweight or obese compared with lean individuals. For example, extended morning fasting versus daily breakfast consumption upregulates the expression of genes involved in lipid turnover in adipose tissue in lean humans but not in humans with obesity (27). Therefore, in order to fully understand the potential for nutrient-exercise timings to alter metabolism, exercise adaptations, and metabolic health in individuals at increased risk of metabolic disease, there is a need to study the most relevant populations, such as individuals classified as overweight or obese (20). Studies have previously examined the acute postprandial responses to carbohydrate-exercise timing in men who are overweight or with obesity (28) and the responses to carbohydrate-exercise timing during 6 weeks of high-intensity interval training in women who are overweight or with obesity (29). However, there is currently a lack of evidence investigating acute intramuscular and chronic (training) responses to altering nutrient-exercise timing in men who are overweight or with obesity. This is important since the metabolic responses to fasting and feeding depend on the mode and intensity of exercise and, potentially, biological sex. It is unknown whether carbohydrate provision before versus after moderate-intensity exercise affects adaptations to exercise training in these populations.

To this end, the aim of the present work was to assess the acute and chronic effects of manipulating nutrient-exercise timing on lipid metabolism, skeletal muscle adaptations, and OGIS in men who are overweight or with obesity. We hypothesized that nutrient-exercise interactions would affect the acute metabolic responses to exercise, with increased whole-body and intramuscular lipid utilization with exercise performed before versus after nutrient provision (mixed-macronutrient breakfast; 65% kcal carbohydrate). We also hypothesized that regular exercise performed before versus after carbohydrate provision would result in greater training-induced increases in OGIS in men classified as overweight or obese.

## Materials and Methods

### Ethical approval

This project comprised 2 experiments. We first assessed the acute metabolic and mRNA responses to manipulating nutrient-exercise timing (Acute Study), followed by a 6-week randomized, controlled trial to assess the longer-term adaptations in response to carbohydrate-exercise timing (Training Study). All participants provided informed written consent prior to participation. Potential participants were excluded if they had any condition or were taking any medication known to alter any of the outcome measures. The studies were registered at https://clinicaltrials.gov (NCT02397304 and NCT02744183, respectively). Protocols were approved by the National Health Service Research Ethics Committee (15/WM/0128 and 16/SW/0260, respectively), and experiments were conducted in accordance with the Declaration of Helsinki.

### Acute study

In the Acute Study, 12 sedentary men classified as overweight or obese were recruited from the Birmingham region of the United Kingdom. The main exclusion criteria included being regularly physically active, having hypertension, or having possible (undiagnosed) T2D. Participant characteristics are shown in Table 1.

**Table 1.** Participant Characteristics

	Study 1	Study 2—Training Study			
Characteristic	Acute Study	CON	CHO-EX	EX-CHO	*P-*value (Training Study)
n	12	9	12	9	
Body mass (kg)	95.1 (13.6)	101.1 (19.5)	95.2 (12.4)	98.0 (18.8)	*0.73*
BMI (kg·m^-2^)	30.2 (3.5)	31.8 (5.8)	30.3 (3.9)	30.8 (4.1)	*0.75*
Waist circumference (cm)	105.7 (11.6)	107.7 (14.8)	103.9 (8.9)	104.7 (11.6)	*0.63*
Hip circumference (cm)	110.9 (6.5)	110.8 (8.4)	111.4 (7.1)	111.6 (8.5)	*0.32*
Waist-to-hip ratio	0.95 (0.08)	0.97 (0.06)	0.93 (0.04)	0.94 (0.05)	*0.31*
V̇O_2_peak (ml·kg^-1^·min^-1^)	29.1 (5.3)	32.6 (7.7)	34.3 (5.6)	32.4 (4.0)	*0.71*
PPO (W)	156 (39)	204 (47)	208 (26)	203 (22)	*0.73*
Physical activity level	-	1.71 (0.16)	1.68 (0.16)	1.68 (0.11)	*0.90*

Data are means (standard deviation) for men classified as overweight or obese.

Abbreviations: BMI, body mass index; V̇O_2_peak, peak oxygen uptake; PPO, peak power output; CON, control; CHO-EX, carbohydrate-exercise; EX-CHO, exercise-carbohydrate.

This was a randomized crossover study where on 1 visit (breakfast-exercise), a standardized breakfast (cornflake cereal with skimmed milk, whole meal toast, sunflower spread, and strawberry jam) was consumed upon arrival at the laboratory (and following 48 hours of diet control). The breakfast provided 25% of estimated daily energy requirements (calculated as resting metabolic rate [RMR] multiplied by a physical activity factor of 1.53 (30)) and was 65% carbohydrate, 20% fat, and 15% protein. After a 90-minute period of rest, 60 minutes of cycling exercise was then performed at 65% peak oxygen uptake (V̇O_2_ peak). Expired gas samples were collected at 25 to 30 minutes and 55 to 60 minutes of exercise to determine whole-body substrate utilization rates. Blood was sampled in the overnight-fasted state, at 45 minutes post breakfast and immediately before exercise was performed (90 minutes post breakfast), every 30 minutes during exercise and at 60-minute intervals during a 3-hour postexercise recovery. In a subset of participants (n = 8) vastus lateralis muscle was sampled pre-exercise and immediately postexercise to assess fiber-type specific intramuscular triglyceride (IMTG) and mixed-muscle glycogen utilization. A third muscle sample (taken at 3 hours postexercise) was used to assess the intramuscular gene expression (mRNA) responses to exercise (n = 7). On the other visit (exercise-breakfast), the participants completed the same protocol, but the breakfast was consumed immediately after the postexercise muscle sample. The primary outcome for the Acute Study was intramuscular lipid utilization during exercise before versus after nutrient ingestion.

### Training study

**Figure 1.**
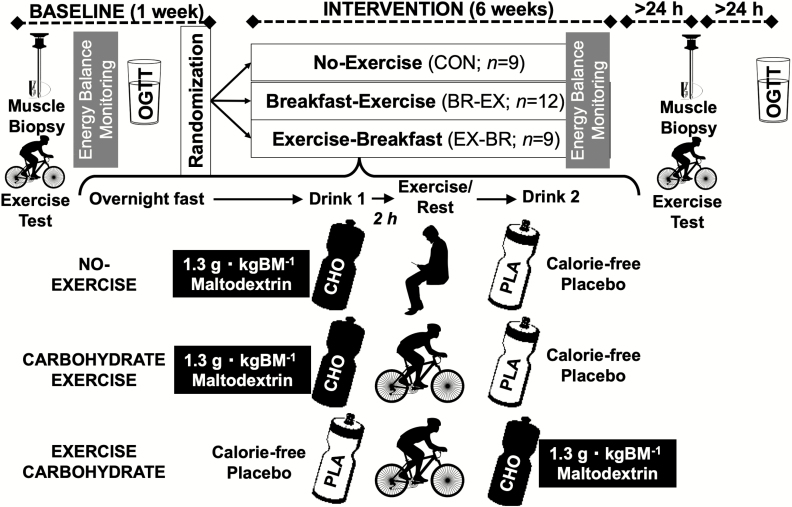
Protocol schematic for the Training Study.

To assess longer-term adaptive (ie, training) responses to altering nutrient- (carbohydrate-) exercise timing (Training Study), we recruited 30 sedentary men who were overweight and obese (self-reported nonexercisers) from the Bath region of the United Kingdom (Table 1). This was a single-blind, randomized, controlled trial with participants allocated to a no-exercise control group (CON; n = 9), a (carbohydrate-only) breakfast before exercise group (CHO-EX; n = 12), or an exercise before (carbohydrate-only) breakfast group (EX-CHO; n = 9) for 6 weeks (Fig. 1). The exercise was supervised moderate-intensity cycling (Monark Exercise AB, Vansbro, Sweden) performed 3 times per week, starting at 50% peak power output [PPO] (weeks 1–3) and increasing to 55% PPO (weeks 4–6). The duration of the exercise sessions progressed from 30 (week 1) to 40 (week 2) to 50 minutes (weeks 3–6). All sessions were supervised at the University of Bath. During every one of the 336 exercise training sessions, 1-minute expired gas samples were collected every 10 minutes to assess substrate utilization and heart rate (Polar Electro Oy, Kempele, Finland), and ratings of perceived exertion (31) were recorded.

Participants ate their evening meal before 2000 hours the evening prior to any exercise sessions. Participants in CHO-EX were given a drink in an opaque bottle made from 1.3 g carbohydrate · kg body mass^-1^ maltodextrin (MyProtein, Northwich, UK) with vanilla flavoring (20% carbohydrate solution) for consumption 2 hours before exercise. They were asked not to eat or drink anything else (except water ad libitum) in this period and confirmed they had consumed the drink before exercising. After exercise, they were provided a taste-matched placebo (water and vanilla flavoring) to consume 2 hours after exercise and were asked not to consume anything else during this period. Participants in EX-CHO were given the same drinks but with the order of the drinks reversed. Participants in CON were given the same drinks for 3 days per week during the intervention, with the carbohydrate drink as breakfast (0800–0900 hours) and the placebo for consumption with their lunch (1100–1300 hours). These participants were asked not to consume anything else between the drinks. There were no other diet controls in the intervention. Blinding of the groups was deemed successful because at exit interview, 25 (83%) participants revealed they could not detect a difference between the carbohydrate and placebo drinks nor could they identify which contained carbohydrate. Five participants determined which drink had carbohydrate (CON, n = 1; CHO-EX, n = 2; EX-CHO, n = 1), but this is within the proportion that could do so at random. Pre- and postintervention, an oral glucose tolerance test (OGTT), a vastus lateralis sample (fasting, rested state), and an exercise test (to assess V̇O_2_ peak and the capacity for lipid utilization during exercise in the fasted state) were undertaken. Postintervention tests were between 24 hours and 48 hours (for muscle sampling) and between 48 hours and 72 hours (for OGTT) after the last exercise training session to reduce any residual effects of the last exercise bout performed on these measurements. The primary outcome for the Training Study was the pre- to postintervention change in the glycemic and insulinemic responses to the OGTT, which were also used to derive an index of OGIS (as described subsequently).

### Pretrial standardizations

For both studies, the participants were asked to maintain their normal physical activity behaviors and to abstain from alcoholic and caffeinated drinks for 24 hours prior to all main laboratory trials. Food intake ceased at a mean [range] of 2000 hours [1900-2100 hours] on the evening before testing, and participants fasted overnight (minimum of 10 hours). For all trials, participants arrived at the laboratory at a mean [range] of 0800 hours [0700-0900 hours], with the exact time replicated for subsequent trials. For the Acute Study, participants were provided with a standardized weight-maintaining diet (50% carbohydrate, 35% fat, 15% protein) based on their estimated energy requirements (RMR multiplied by the physical activity factor of 1.53, as stated previously) for consumption for 48 hours prior to main trials. For the Training Study, they recorded the composition of their evening meal on the day before in a preintervention trial and replicated this meal for the postintervention trial, in line with guidelines for testing postprandial glycemic control (32). This protocol produces fasting muscle and liver glycogen and fasting intramuscular lipid concentrations that are consistent across trial days (33).

### Anthropometry

Stature was measured to the nearest 0.1 cm using a stadiometer (Seca Ltd, Birmingham, UK). Body mass was measured to the nearest 0.1 kg using electronic weighing scales (Acute Study: OHaus Champ II Scales, Parsippany, NJ, USA; Training Study: BC543 Monitor, Tanita, Japan). Waist and hip circumferences were measured to the nearest 0.1 cm and according to the World Health Organization guidelines.

### Exercise tests

Participants completed exercise tests on an electronically braked ergometer. In the Acute Study, the starting intensity was 35 Watts (W), and this was increased by 35 W every 3 minutes until volitional exhaustion. In the Training Study, the starting intensity for the exercise test was 50 W, which was increased by 25 W every 3 minutes. Heart rate (Polar Electro Oy, Kempele, Finland) and continuous breath-by-breath measurements were recorded (Acute Study: Oxycon Pro, Jaeger, Wurzburg, Germany; Training Study: TrueOne2400, ParvoMedics, Sandy, USA). Volume and gas analyzers were calibrated using a 3-L calibration syringe (Hans Rudolph, Kansas City, USA) and a calibration gas (16.04% O_2_, 5.06% CO_2_; BOC Industrial Gases, Linde AG, Germany). PPO was calculated as the work rate of the final completed stage plus the fraction of time in the final, noncompleted stage, multiplied by the W increment. V̇O_2_ peak was the highest measured V̇O_2_ over a 30-second period, using methods and attainment criteria previously reported (34).

### Blood sampling and analysis

In the Acute Study, 10 mL of blood was sampled from an antecubital forearm vein and 6 mL dispensed into ethylenediaminetetraacetic acid-coated tubes (BD, Oxford, UK) and centrifuged (4°C at 3500 rpm) for 15 minutes (Heraeus Biofuge Primo R, Kendro Laboratory Products Plc., UK). Resultant plasma was dispensed into 0.5 mL aliquots and frozen at –20°C before longer-term storage at –80°C. A proportion of the sample (4 mL) was allowed to clot for serum in a plain vacutainer prior to centrifugation. Samples were analyzed for plasma glucose, glycerol, and non-esterified fatty acid (NEFA) using an ILAB 650 Clinical Chemistry Analyzer (Instrumentation Laboratory, Warrington, UK). Serum insulin concentrations were measured with an ELISA kit (Invitrogen; Cat#KAQ1251) and Biotek ELx800 analyzer (Biotek Instruments, Vermont, USA).

In the Training Study, prior to blood sampling, participants placed their dominant hand into a heated-air box set to 55°C. After 15 minutes of rest, a catheter was placed (retrograde) into a dorsal hand vein and 10 mL of arterialized blood was drawn for a baseline sample (overnight-fasted state) (35). Then a 75-g OGTT was completed and arterialized blood sampled every 15 minutes for 2 hours and processed (as detailed above) for plasma. Plasma glucose (intra-assay coefficient of variation [CV]: 2.50%), glycerol, triglyceride (glycerol-blanked), and total high-density lipoprotein– cholesterol and low-density lipoprotein–cholesterol concentrations were measured using an automated analyzer (Daytona; Randox Lab, Crumlin, UK). Plasma insulin (Mercodia AB; reference #10-1113-01) and C-peptide (Sigma Aldrich; reference #EZHCP-20K) concentrations were measured using commercially available ELISA kits (intra-assay CV for insulin: 3.86% and for C-peptide: 4.26%). NEFA concentrations were assessed via an enzymatic colorimetric kit (WAKO Diagnostics; references #999–34691/#991–34891; intra-assay CV: 7.95%). All analysis was done in batch and for a given participant all samples were included on the same plate.

### Muscle sampling

All vastus lateralis skeletal muscle samples were collected under local anesthesia (~5 mL 1% lidocaine, Hameln Pharmaceuticals Ltd., Brockworth, UK) and from a 3 to 6 mm incision at the anterior aspect of the thigh using a 5-mm Bergstrom biopsy needle technique adapted for suction. For the Acute Study, samples were collected pre-exercise and immediately postexercise and at 3 hours postexercise. To enable the analysis of the IMTG content, approximately 15 to 20 mg of each sample were embedded in Tissue-Tek OCT (Sigma Aldrich, Dorset, UK) on cork disc and frozen in liquid nitrogen-cooled isopentane before being transferred into an aluminium cryotube and stored at –80°C. Remaining muscle (for glycogen and gene expression analysis) was frozen in liquid nitrogen and stored at –80°C. In the Acute Study, the pre-exercise and immediately postexercise muscle samples were used to measure IMTG content and muscle glycogen concentrations (36). The mRNA expression of 34 metabolic genes was analyzed using a custom RT2 Profiler PCR Array (Qiagen, Germantown, MD, USA) using the pre-exercise and 3-hour postexercise samples (36).

For the Training Study, samples were collected pre- and postintervention with participants in a fasted, resting state, with both samples from their dominant leg. Muscle was extracted from the needle and frozen in liquid nitrogen before storage at –80°C. Frozen wet muscle (80–100 mg) was freeze dried and powdered, with visible blood and connective tissue removed. Ice cold lysis buffer (50 mM Tris [pH 7.4], 150 mM NaCl, 0.5% sodium deoxycholate; 0.1% sodium dodecyl sulfate (SDS) and 0.1% NP-40) with protease and phosphatase inhibitors was added. Samples were homogenized with a dounce homogenizer before 60 minutes incubation (4°C with rotation) and 10 minutes centrifugation (4°C and 20 000 g [relative centrifugal force]). The protein content of the resultant supernatant was measured using a bicinchoninic acid assay. In the Training Study, western blotting was used to measure the content of proteins involved in glucose transport, insulin signaling, and lipid metabolism (OXPHOS, CPT-1, CD36, GLUT4, CHC22, CHC17, AMPKα, Akt, AS160). The methods used have been previously described (18), and all supplementary material and figures are located in a digital research materials repository (36). The phospholipid composition of skeletal muscle samples was measured by gas-liquid chromatography (36). Citrate synthase activity was measured using a commercially available assay (Abcam: reference #ab119692).

### Energy expenditure and intake (Training Study)

Average daily energy expenditure was calculated as the sum of the RMR, diet-induced thermogenesis (10% of self-reported daily energy intake), and PAEE. To assess RMR, participants rested in a semi-supine position for 15 minutes before four 5-minute expired air samples were collected (37). The participants were provided with the mouthpiece 1 minute prior to sample collections (as a stabilization period), which were collected into a 200-L Douglas bag (Hans Rudolph, Kansas City, USA) via falconia tubing (Baxter, Woodhouse and Taylor Ltd, Macclesfield, UK). Concurrent measures of inspired air were also made to correct for changes in the ambient O_2_ and CO_2_ concentrations. Expired O_2_ and CO_2_ concentrations were measured in a volume of each sample using paramagnetic and infrared transducers (Mini HF 5200, Servomex Group Ltd., Crowborough, UK). The sensor was calibrated with low (0% O_2_ and 0% CO_2_) and high (16.04% O_2_ and 5.06% CO_2_) calibration gases (BOC Industrial Gases, Munich, Germany). Substrate utilization rates were calculated via stochiometric equations (38, 39). Energy expenditure was calculated assuming that fatty acids, glucose, and glycogen provide 40.81 kJ·g^-1^, 15.64 kJ·g^-1^, and 17.36 kJ·g^-1^ of energy, respectively. To measure free-living PAEE, participants wore an Actiheart^TM^ (combined heart rate-accelerometery) for 7 days (Cambridge Neurotechnology, Papworth, UK) (40–42). Energy expenditure and heart rate values from rest and exercise were entered in the Actiheart software for an individually calibrated model. Participants were asked to keep a written record of their food and fluid intake for 4 days over a typical 7-day period (including a weekend day) before and during the last week of the intervention. Weighing scales were provided to increase the accuracy of records. Records were analyzed using Nutritics software (Nutritics Ltd., Dublin, Ireland). The macronutrient composition of each food was taken from the manufacturer’s labels, but if this was not possible, foods were analyzed via the software database, or comparable brands were used to provide the relevant information (kept constant across records).

### Statistics

In the Acute Study, the sample size was based upon data demonstrating an attenuation of intramuscular lipid utilization during exercise with carbohydrate intake before and during exercise with an effect size of *d* = 1.5. (21) We aimed to recruit 12 participants assuming at least 8 participants would complete the study with biopsies to provide >90% power with α set at 0.05. In the Training Study, a sample size estimation was completed using data from a training study in healthy, lean men (25). In that study, a change in the plasma glucose area under the curve (AUC) for an OGTT mean [SD] of –65 [53] mmol·min·L^-1^ was shown in an EX-CHO group versus a mean [SD] of +21 [47] mmol·min·L^-1^ for a CON group. With α set at 0.05, 9 participants were required for a >90% chance of detecting this effect. We recruited 30 participants to account for the possibility of an unequal allocation of participants across 3 groups when using a stratified randomization schedule. Participants were allocated to the CON (n = 9), CHO-EX (n = 12), or EX-CHO (n = 9) groups using this schedule, which was generated by an author who was not involved in trial days and included a factor for Physical Activity Level (PAL) and the time-averaged glucose AUC for the baseline OGTT, which was assessed using a Freestyle Freedom Lite Glucose Meter. This was to ensure an even distribution of less (PAL < 1.65) and more active (PAL > 1.65) participants and participants with glucose AUC values above or below 8 mmol·L^-1^.

Data are presented as means (± 95% confidence intervals), except for participant characteristics (which are mean ± SD). A Shapiro-Wilk test was performed to test for normal distribution and if this was not obtained, nonparametric tests (eg, Wilcoxon matched-pairs signed-rank tests) were employed. In the Acute Study, differences between groups were assessed with paired t*-*tests or a two-way repeated measures for analysis of variance (ANOVA; for variables dependent on time). In the Training Study, one-way ANOVAs were used to assess differences between groups at baseline and two-way mixed-design ANOVAs were used to assess differences between groups in response to the intervention (group x time). If interaction effects were identified, independent t-tests were used to locate variance, with Holm-Bonferroni step-wise adjustments made. Correlations between variables were explored using Pearson r correlation or Spearman rank R for normal or nonnormal distributions, respectively. A significance level of *P* < 0.05 was always used. The area under the concentration-time curve (AUC) was calculated via the trapezoid rule and divided by the duration of an observation period of interest for a time-averaged summary value. Plasma glucose and insulin concentrations were used to assess OGIS (OGIS index; as per instructions provided at http://webmet.pd.cnr.it/ogis/) (43). Statistical analyses were completed on IBM SPSS statistics V22 (IBM, Armonk, NY, USA) for windows (except for the Holm-Bonferroni adjustments, which were completed on Microsoft Excel), and GraphPad Prism V7 (GraphPad, San Diego, CA, USA) was used to prepare the figures. As we were unable to collect data from all participants for all measured outcomes, the *n* are always displayed in all figure and table captions.

## Results

Exercise before nutrient ingestion increases whole-body and skeletal muscle lipid utilization but does not differentially modulate muscle gene expression.

**Figure 2.**
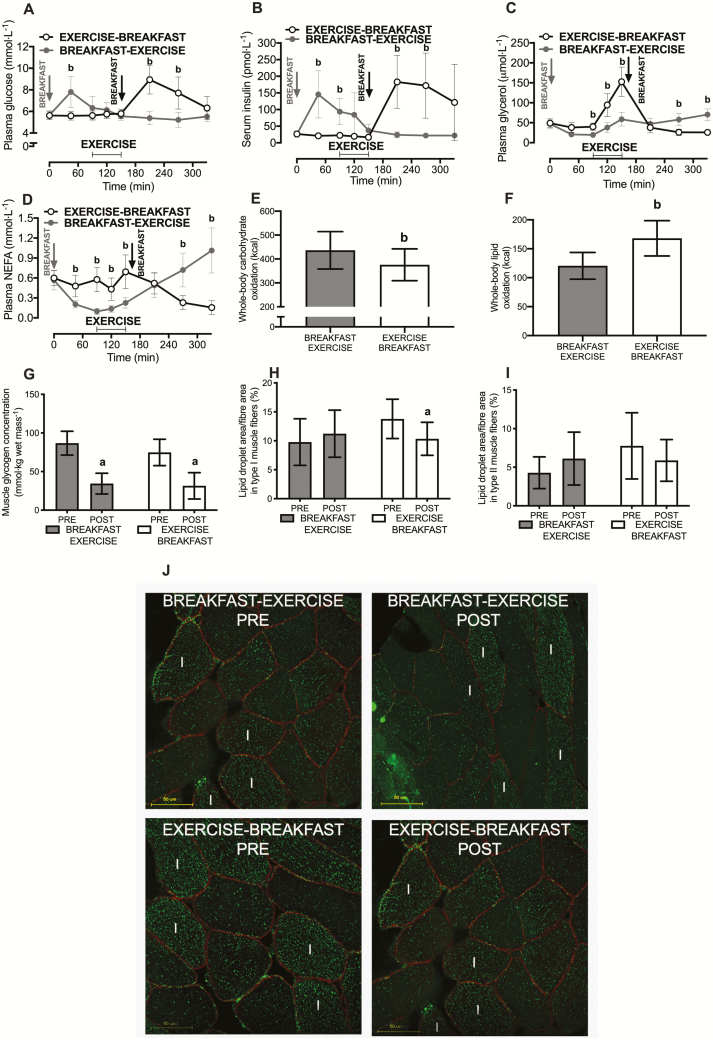
Plasma glucose (**A**), serum insulin (**B**), plasma glycerol (**C**), and plasma NEFA (**D**) concentrations and whole-body carbohydrate (**E**) and lipid (**F**) utilization rates. Muscle was sampled pre-exercise and immediately postexercise (vastus lateralis) to assess mixed-muscle glycogen (**G**) and fiber-type specific intramuscular lipid (IMTG) utilization (**H & I**). Panel **J** shows representative images from IMTG staining where IMTG (stained green) in combination with dystrophin (to identify the cell border and stained red) is shown from skeletal muscle samples of a representative participant for the carbohydrate-exercise and exercise-carbohydrate trials. White I shows type 1 fibers, and all other fibers are assumed to be type II. Yellow bars are scale (50 μm). All data are presented as means ± 95% confidence interval. For panels **A–F,** n = 12 men classified as overweight or obese; for panels **G**, **H, and I,** n = 9. The difference between PRE versus POST exercise is designated by ***a***; and the difference between BREAKFAST-EXERCISE versus EXERCISE-BREAKFAST is designated by ***b***. (*P* < 0.05).

In the Acute Study, exercising before versus after nutrient provision increased the acute plasma glucose and serum insulin responses to food consumption (Fig. 2A and B). The plasma glucose AUC was 6.70 [6.00 to 7.39] mmol·L^-1^·330 min^-1^ with exercise before nutrient provision versus 5.91 [5.33 to 6.50] mmol·L^-1^·330 min^-1^ with exercise after nutrient provision (*P* < 0.01). The serum insulin AUC was 86.9 [48.5 to 125.2] pmol·L^-1^·330 min^-1^ with exercise before nutrient provision versus 55.3 [31.2 to 79.3] pmol·L^-1^·330 min^-1^ with exercise after nutrient provision (*P* < 0.01). Exercise performed before versus after nutrient provision resulted in higher glycerol and NEFA concentrations during the exercise (Fig. 2C and D).

Nutrient provision before exercise potently altered whole-body metabolism, resulting in an increase in whole-body carbohydrate utilization (Fig. 2E) and a decrease in whole-body lipid utilization (Fig. 2F). In skeletal muscle, glycogen utilization during exercise (time effect, *P* < 0.01) was independent of nutrient-exercise timing (time x trial interaction effect *P* = 0.12; Fig. 2G). However, the type I muscle fiber IMTG content was only reduced with exercise performed before nutrient provision (time x trial interaction: *P* = 0.02; Fig. 2H and J). A similar pattern was observed for the type II muscle fiber IMTG content (time x trial interaction: *P* = 0.04; Fig. 2I and J), although the reduction with exercise before nutrient provision did not achieve statistical significance after post hoc corrections. Nonetheless, clear differences (both *P* < 0.05) in the net changes in the IMTG content were observed in both fiber types with exercise before versus after nutrient provision (for type I: –3.44% [–1.61 to –5.26] versus 1.44% [–1.46 to 4.34] area covered by lipid staining and type II: –1.89% [–0.16 to –3.61] versus 1.83% [0.50 to 3.17] area covered by lipid staining for exercise before versus after nutrient provision, respectively).

**Figure 3.**
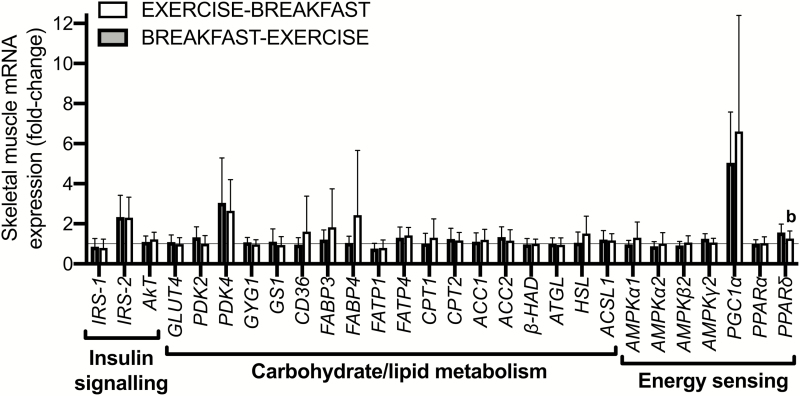
Skeletal muscle mRNA expression responses to a single bout of exercise before versu*s* after nutrient provision (in the form of breakfast) in overweight men (n = 8). Muscle was sample pre-exercise and at 3 hours postexercise (vastus lateralis) to assess the intramuscular gene expression responses to exercise. The difference between EXERCISE-BREAKFAST vs BREAKFAST-EXERCISE. (*P* < 0.05).

Of the 34 selected genes that are implicated in metabolic adaptations to exercise, only 8 genes were altered by exercise, whereby *IRS-1* and *FATP1* were decreased postexercise compared to baseline (*P* < 0.05), and *IRS-2, PDK4, PGC1α, FATP4*, and *ACSL1* were increased postexercise compared to baseline (all *P* < 0.05). However, only *PPARδ* was differentially expressed by nutrient-exercise timing and was higher with breakfast before versus after exercise (*P* < 0.05; Fig. 3).

### Exercise training before carbohydrate provision leads to sustained increases in lipid utilization

In the Training Study, the compliance to the training was 100%, as all sessions were completed as prescribed. The average [SD] exercise intensity was 62% [5] V̇O_2_ peak in CHO-EX and 62% [4] V̇O_2_ peak in EX-CHO (*P* = 0.98), and the heart rate response and average rating [SD] of perceived exertion to the exercise training were 140 [13] versus 134 [8] beats·min^-1^ in CHO-EX versus EX-CHO (*P* = 0.18) and 13 [1] arbitrary units versus 13 [1] arbitrary units (6–20 rating scale) in CHO-EX versus EX-CHO; (*P* = 0.54), respectively.

**Figure 4.**
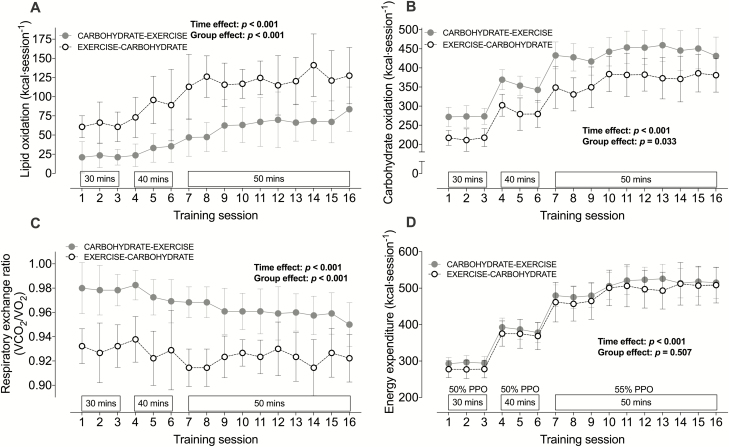
Whole-body rates of lipid utilization (**A**), carbohydrate utilization (**B**), the respiratory exchange ratio (**C**), and energy expenditure (**D**) during every exercise session in a 6-week training intervention with exercise performed after (CARBOHYDRATE-EXERCISE) or before carbohydrate intake (EXERCISE-CARBOHYDRATE). All data are presented as means ± 95% confidence interval. For control, n = 9; for carbohydrate-exercise, n = 12; and for exercise-carbohydrate, n = 9 men classified as overweight or obese.

In the Training Study, rates of whole-body lipid utilization were around 2-fold higher with exercise before versus after carbohydrate provision, and this difference between the conditions was sustained throughout the whole 6-week intervention (Fig. 4A). As a consequence, regular exercise before (versus after) carbohydrate provision increased cumulative whole-body lipid utilization (during exercise) over a 6-week intervention, from 799 kcal (530 to 1069 kcal) in CHO-EX to 1666 kcal (1260 to 2072 kcal) in EX-CHO (*P* < 0.01). This was accompanied by a decrease in rates of whole-body carbohydrate utilization during exercise (Fig. 4B), as reflected by a decrease in the respiratory exchange ratio (group effect, *P* < 0.01; Fig. 4C). However, cumulative energy expenditure throughout the exercise intervention did not differ with exercise performed before versus after carbohydrate provision (Fig. 4D; 7207 kcal [6739 to 7676 kcal] in CHO-EX versus 6951 kcal [6267 to 7635 kcal] in EX-CHO; *P* = 0.48).

### Exercise training before versus after carbohydrate provision increases an index of oral glucose insulin sensitivity

**Figure 5.**
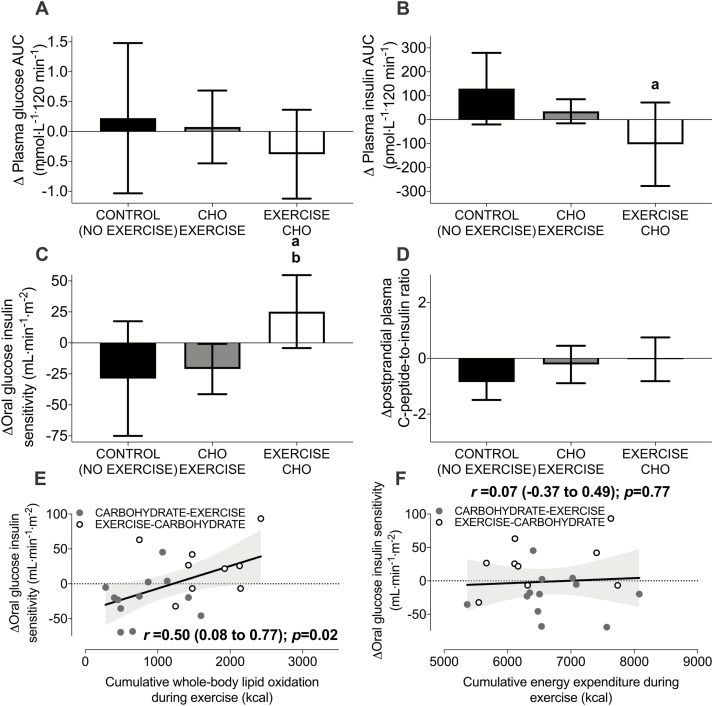
The change in the plasma glucose area under the curve (AUC; **A**), the change in the plasma insulin AUC (**B**), the change in the oral glucose insulin sensitivity index (OGIS; **C**), and the change in the postprandial plasma C-peptide: insulin ratio (**D**) in response to a 6-week training intervention with exercise performed after (CARBOHYDRATE-EXERCISE) or before carbohydrate intake (EXERCISE-CARBOHYDRATE). Panels **E** and **F** display Pearson correlations between changes in the OGIS index and cumulative lipid utilization and energy expenditure throughout the exercise training intervention, respectively. All data are presented as means ± 95% confidence interval. For control, n = 9; for carbohydrate-exercise, n = 12; and for exercise-carbohydrate, n = 9 men classified as overweight or obese. The shaded grey area represents the 95% confidence bands for the regression line. The difference between CONTROL versus EXERCISE-CARBOHYDRATE is designated by ***a***. The difference between CARBOHYDRATE-EXERCISE versus EXERCISE-CARBOHYDRATE is designated by ***b*** (*P* < 0.05).

The OGTT-derived estimate of peripheral insulin sensitivity (the OGIS index; *P* = 0.26), postprandial glycemia (*P* = 0.80), and postprandial insulinemia (*P* = 0.30) were similar between groups pre-intervention (36). The intervention-induced changes in postprandial glycemia (time x group interaction, *P* = 0.54; Fig. 5A), and fasting blood lipid profiles were unaffected by carbohydrate-exercise timing [pre- and postintervention data are shown in Table 2 (36). However, exercise training before, but not after carbohydrate intake reduced postprandial insulinemia (time x group interaction *P* = 0.03; Fig. 5B). Exercise training before versus after carbohydrate provision also increased the OGIS index (time x group interaction *P* = 0.03; Fig. 5C) (37). The plasma C-peptide-to-insulin ratio was not differentially altered by carbohydrate-exercise timing (time x group interaction, *P* = 0.12; Fig. 5D). The change in the OGIS index in response to exercise training was positively and moderately correlated with cumulative lipid utilization during exercise throughout the intervention (Fig. 5E) but not with cumulative energy expenditure (Fig. 5F).

**Table 2.** Fasting Plasma Metabolite and Hormone Concentrations for the Control (CON; n** = **9), Carbohydrate-Exercise (CHO-EX; n** = **12) and Exercise-Carbohydrate (EX-CHO; n** = **9) Groups

Metabolite/Hormone	Pre-Intervention	Post-Intervention	Δ From Pre-Intervention	*Time x Group Interaction*
CON glucose (mmol·L^-1^)	5.39 (0.49)	5.57 (0.66)	0.17 (–0.32, 0.66)	*F* = 1.413 *P* = 0.26
CHO-EX glucose (mmol·L^-1^)	5.48 (0.33)	5.60 (0.47)	0.12 (–0.30, 0.54)	
EX-CHO glucose (mmol·L^-1^)	5.72 (0.71)	5.46 (0.72)	–0.27 (–0.67, 0.13)	
CON insulin (pmol·L^-1^)	95 (121)	81 (63)	–14 (–7, 8)	F = 0.327 *P* = 0.72
CHO-EX insulin (pmol·L^-1^)	43 (23)	43 (24)	0 (–14, 15)	
EX-CHO insulin (pmol·L^-1^)	49 (43)	47 (35)	–2 (–20, 14)	
CON HOMA-IR (au)	0.35 (0.04)	0.34 (0.03)	–0.01 (–0.02, 0.00)	F = 0.458 *P* = 0.40
CHO-EX HOMA-IR (au)	0.37 (0.03)	0.37 (0.04)	0.00 (–0.01, 0.01)	
EX-CHO HOMA-IR (au)	0.37 (0.04)	0.37 (0.04)	0.01 (–0.02, 0.04)	
CON NEFA (mmol·L^-1^)	0.36 (0.16)	0.39 (0.11)	0.03 (–0.10, 0.15)	F = 1.021 *P* = 0.37
CHO-EX NEFA (mmol·L^-1^)	0.44 (0.15)	0.39 (0.10)	–0.05 (–0.11, 0.15)	
EX-CHO NEFA (mmol·L^-1^)	0.34 (0.09)	0.32 (0.10)	–0.02 (–0.09, 0.05)	
CON TAG (mmol·L^-1^)	1.59 (0.77)	2.05 (0.70)	0.46 (–0.06, 0.98)	F = 5.967 *P* < 0.01
CHO-EX TAG (mmol·L^-1^)	1.34 (0.84)	1.11 (0.42)	–0.24 (–0.55, 0.76)^a^	
EX-CHO TAG (mmol·L^-1^)	1.10 (0.31)	0.88 (0.36)	–0.22 (–0.41, –0.03)^b^	
CON cholesterol (mmol·L^-1^)	4.41 (1.23)	4.59 (1.36)	0.19 (–0.74, 1.12)	F = 1.707 *P* = 0.20
CHO-EX cholesterol (mmol·L^-1^)	3.77 (1.34)	3.72 (1.21)	–0.05 (–0.41, 0.30)	
EX-CHO cholesterol (mmol·L^-1^)	3.74 (0.82)	3.24 (0.92)	–0.51 (–0.95, –0.07)	
CON LDL cholesterol (mmol·L^-1^)	3.36 (1.33)	3.56 (1.36)	0.20 (–0.50, 0.89)	F = 2.110 *P* = 0.14
CHO-EX LDL cholesterol (mmol·L^-1^)	2.71 (0.97)	2.70 (0.99)	–0.01 (–0.31, 0.28)	
EX-CHO LDL cholesterol (mmol·L^-1^)	2.72 (0.57)	2.31 (0.59)	–0.41 (–0.80, –0.02)	
CON HDL cholesterol (mmol·L^-1^)	0.84 (0.17)	0.86 (0.20)	0.02 (–0.14, 0.18)	F = 1.634 *P* = 0.21
CHO-EX HDL cholesterol (mmol·L^-1^)	0.82 (0.31)	0.84 (0.33)	0.02 (–0.05, 0.10)	
EX-CHO HDL cholesterol (mmol·L^-1^)	0.90 (0.23)	0.81 (0.26)	–0.09 (–0.19, 0.01)	

Data are means and (standard deviation) except for change scores, which are means and (95% confidence interval).

Abbreviations: HOMA-IR, the homeostatic model of insulin resistance; NEFA, non-esterified fatty acid; TAG, triglyceride, HDL, high-density lipoprotein; LDL, low-density lipoprotein.

^a^ Difference in change from pre- to postintervention for CON versus CHO-EX with *P* < 0.05.

^b^ Difference in change from pre- to postintervention for CON versus EX-CHO with *P* < 0.05.

### Carbohydrate-exercise timing does not differentially alter body composition or oxidative capacity

**Figure 6.**
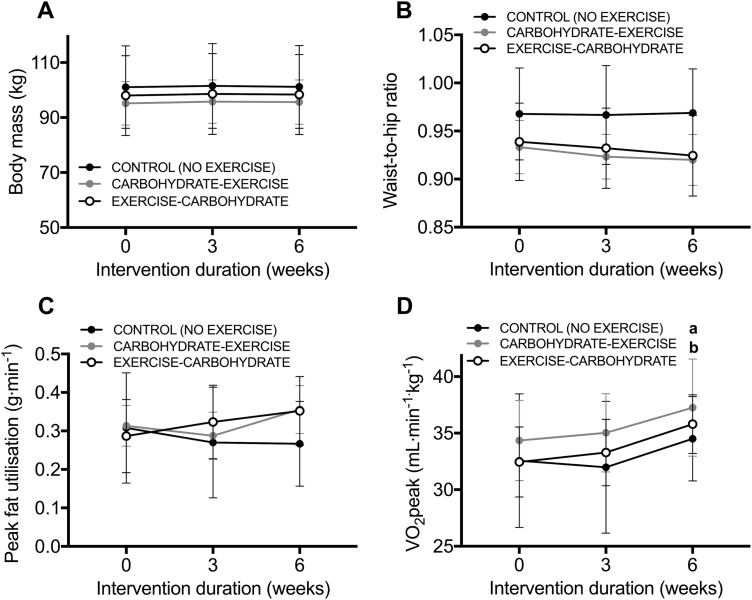
Body mass (**A**), the waist-to-hip ratio (**B**), peak fat utilization rates during an incremental exercise test (**C**), and (**D**) whole-body oxidative capacity (VO_2_peak) at baseline, week 3, and week 6 of an intervention in control (no-exercise), carbohydrate-exercise, and exercise-carbohydrate groups. All data are presented as means ± 95% confidence interval. For control, n = 9; for carbohydrate-exercise, n = 12; and for exercise-carbohydrate, n = 9 men classified as overweight or obese. The difference between CONTROL versus CARBOHYDRATE-EXERCISE is designated by ***a***. The difference between CONTROL versus EXERCISE-CARBOHYDRATE is designated by ***b*** (*P* < 0.05).

Exercise before versus after carbohydrate provision resulted in comparable changes in body mass (time x group interaction, *P* = 0.97; Fig. 6A), a marker of central adiposity (the waist-to-hip ratio; time x group interaction, *P* = 0.17, Fig. 6B), and the peak capacity for whole-body lipid utilization (time x group interaction, *P* = 0.14; Fig. 6C). Exercise training increased V̇O_2_ peak by approximately 3 mL⋅kg⋅min^-1^ relative to a CON group (time x group interaction, *P* = 0.01), but the magnitude of this increase in cardiorespiratory fitness was unaffected by carbohydrate-exercise timing (*P* = 0.54 with carbohydrate-exercise versus exercise-carbohydrate). Self-reported daily energy intake was unaffected by exercise or carbohydrate-exercise timing [time x group interaction, *P* = 0.38; (36)], and although daily energy expenditure was increased in the exercise groups versus control group [time x group interaction, *P* = 0.01; (36)], this increase was unaffected by carbohydrate-exercise timing (*P* = 0.38).

### Effects of carbohydrate-exercise timing on skeletal muscle phospholipids

**Figure 7.**
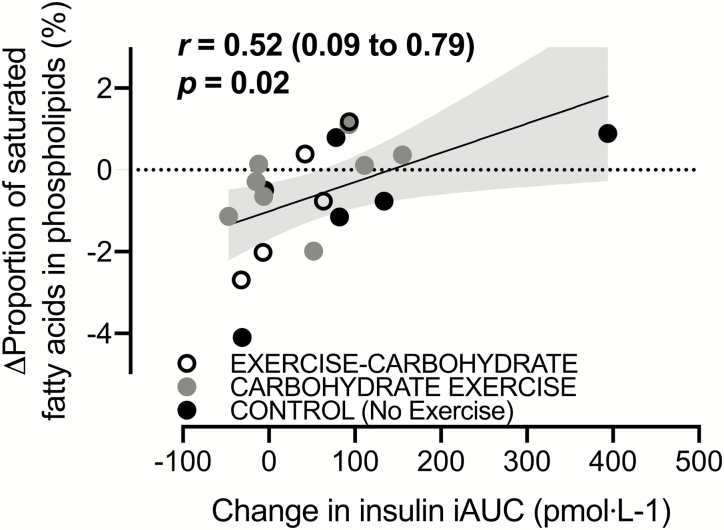
A Pearson correlation between postprandial insulinemia with the change in the proportion of saturated fatty acids in skeletal muscle phospholipids. All data are presented as means ± 95% confidence interval. For control, n = 6; for carbohydrate-exercise, n = 9; and for exercise-carbohydrate, n = 5 men classified as overweight or obese. The shaded area represents the 95% confidence bands for the regression line.

No clear time x group interaction effects were determined for any of the measured fatty acid species except for the proportion of 18:0, which increased with exercise before carbohydrate provision compared to the control group (36). The change in the overall saturated fatty acid content of skeletal muscle phospholipids was moderately and positively correlated with changes in postprandial insulinemia, and the relationship was robust to the exclusion of any single data point (Fig. 7).

### Exercise training before carbohydrate provision augments intramuscular adaptations

**Figure 8.**
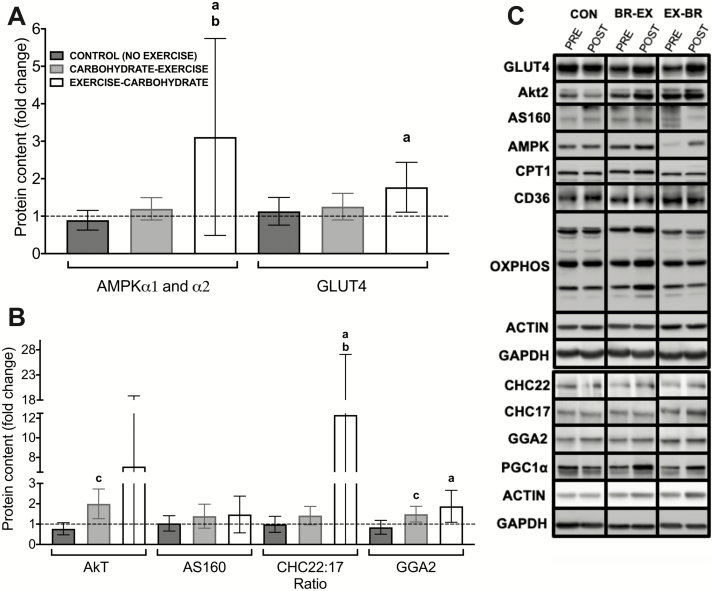
Pre- to postintervention changes in the levels of energy-sensing proteins and proteins involved in insulin-sensitive GLUT4 trafficking in skeletal muscle (**A** and **B**). Representative immunoblots are shown (**C**) for each protein (including those reported in text but not shown in this figure) from the same representative participant as well as the loading controls used. All data are presented as means ± 95% confidence interval, and the dotted horizontal line represents the baseline (pre-intervention) values. For control, n = 6; for carbohydrate-exercise, n = 9; and for exercise-carbohydrate, n = 5 men classified as overweight or obese. The difference between CONTROL versus EXERCISE-CARBOHYDRATE is designated by ***a***; the difference between CARBOHYDRATE-EXERCISE versus EXERCISE-CARBOHYDRATE is designated by ***b***; and the difference between CONTROL versus CARBOHYDRATE-EXERCISE is designated by ***c*** (*P* < 0.05).

Skeletal muscle activated protein kinase (AMPK) protein levels increased approximately 3-fold with exercise training performed before, but not after, carbohydrate provision versus a no-exercise control group (Fig. 8A). However, these increases did not translate into differential changes in proteins including CD36 and CPT-1, which are involved in fatty acid transport in skeletal muscle [both *P* > 0.05; data available online (36)], or markers of mitochondrial oxidative capacity, including the protein levels of the OXPHOS complexes [all *P* > 0.05; data available online (36)] or citrate synthase activity (change from baseline: –2.1 μmol⋅min^-1^⋅mg of protein^-1^ [–12.9 to 8.7] in CON, 7.6 μmol⋅min^-1^⋅mg of protein^-1^ [–1.2 to 16.4] in CHO-EX, and 6.5 μmol⋅min^-1^⋅mg of protein^-1^ [0.2 to 12.8] in EX-CHO; *P* > 0.05). There were also no differential changes in the content of insulin signaling proteins such as Akt2 or AS160 in response to carbohydrate-exercise timing (*P* > 0.05; Fig. 8B). However, there was an approximate 2-fold increase in skeletal muscle GLUT4 protein levels with exercise training performed before (*P* = 0.04), but not after carbohydrate provision (*P* = 0.58) versus a non-exercise control group (Fig. 8A). There was also an increase in the protein levels of the CHC22 clathrin isoform and its associated adaptor protein (GGA2) relative to the CHC17 clathrin isoform, with exercise before versus after carbohydrate provision (both *P* < 0.05; Fig. 8B). When we examined the CHC22 isoform alone [data not shown but available online (36)], we noted baseline differences that may have confounded the interpretation of these fold changes due to regression to the mean. We thus present the CHC22/CHC17 ratio (Fig. 8B) to reflect GLUT4-associated clathrin-mediated membrane traffic relative to total clathrin-mediated membrane traffic.

## Discussion

This is the first study to investigate the effect of nutrient-exercise interactions on key aspects of metabolic health in people classified as overweight or obese during moderate-intensity exercise training. We found that a single exercise bout performed before versus after a mixed-macronutrient meal (65% of kcal carbohydrate) increased whole-body and skeletal-muscle lipid utilization. We then used a 6-week training program to reveal sustained, 2-fold increases in lipid utilization that were maintained throughout 6 weeks of exercise training performed before versus after carbohydrate ingestion. An OGTT-derived estimate of peripheral insulin sensitivity (the OGIS index) increased with exercise training before versus after nutrient provision (albeit with no within-group changes), and this was associated with increased lipid utilization during the exercise training intervention. Exercise training prior to carbohydrate provision also augmented remodeling of phospholipids and increased the levels of energy sensing (ie, AMPK) and glucose transport proteins (ie, GLUT4) in exercised skeletal muscle. These results indicate that nutrient-exercise timing modulates training responsiveness in men who are overweight and links lipid utilization during exercise to training-induced changes in key aspects of metabolic health.

First, we showed that a single bout of exercise performed before versus after nutrient intake increased whole-body lipid utilization (Acute Study). A blunting of IMTG utilization has been shown in type I fibers of lean, healthy men in response to carbohydrate ingestion before and during exercise, compared to exercise in the fasted state (21). Here we demonstrated for the first time that exercise before versus after (a carbohydrate-rich) breakfast increases net IMTG utilization in men classified as overweight or obese. While the authors do acknowledge that absolute IMTG content may have been underestimated due to the analytical procedures used to estimate IMTG (ie, use of Triton X-100 (Sigma-Aldrich, Gillingham, Dorset, UK) detergent, overnight drying of mounting medium), all samples were treated consistently. We also showed that net skeletal muscle glycogen utilization and acute skeletal muscle mRNA responses were largely unaffected by the same exercise performed before versus after the breakfast. This is important because muscle glycogen availability can alter muscle adaptations to exercise training (26). Lower muscle glycogen concentrations are therefore unlikely to have driven the training responses we observed in the Training Study with the present method of nutrient-exercise timing.

Altering substrate availability can also drive adaptive responses to exercise partly by modulating acute mRNA expression in exercised skeletal muscle (44). However, in the present study, only 1 measured gene was differentially expressed in response to exercise before versus after a mixed-macronutrient, carbohydrate-rich breakfast. Specifically, we observed less of an exercise-induced increase in skeletal muscle *PPARδ* expression with exercise before versus after nutrient provision, which is surprising given that *PPARδ* has been implicated in adaptations relating to oxidative capacity and lipid utilization (45). However, previous research has also shown no differential increase in *PPARδ* expression in skeletal muscle when exercise was performed with carbohydrate consumption before/during exercise versus exercise in the fasted state (46). The different response observed in the present study might be because we assessed the effect of nutrient-exercise timing (ie, nutrient provision before versus after exercise) rather than the omission versus ingestion of nutrients. While it is possible that some changes in mRNA expression were missed due to the timing of muscle biopsies, this result suggests that inferences cannot necessarily be extrapolated from studies assessing the effects of nutrient ingestion versus nutrient omission to inform responses to models of nutrient-exercise timing.

In the Training Study, we then showed that the acute increases in whole-body lipid utilization during a single bout of exercise performed before versus after nutrient intake were sustained throughout 6 weeks of exercise training. Moreover, only exercise training performed before carbohydrate ingestion reduced postprandial insulinemia and increased the OGGT-derived estimate of peripheral insulin sensitivity (ie, the OGIS index). As the plasma C-peptide-to-insulin ratio was not differentially altered by nutrient-exercise timing, the reduction in postprandial insulinemia with exercise performed before versus after carbohydrate ingestion is likely to be due to a reduction in insulin secretion rather than an increase in hepatic insulin extraction (47). It should also be noted that difference between the exercise groups for the change in the OGIS index was also broadly equivalent to the difference between individuals classified as having a healthy phenotype compared to individuals with impaired glucose tolerance (48). It has previously been reported that nutrient-exercise timing does not alter the adaptive postprandial response to high-intensity (~90% of maximum heart rate) interval training in women who are overweight or with obesity (29) or alter fasting glycemia or insulinemia in people with T2D (49). However, during exercise at (or above) 75% V̇O_2_peak, feeding status does not alter fat oxidation during exercise since fat oxidation rates are suppressed by the high exercise intensity (50). Therefore, when the present data are taken in light of these previous findings, it is likely that nutrient timing is more important for driving exercise-induced adaptations in postprandial metabolism at moderate rather than high-exercise intensities.

Skeletal muscle phospholipid composition is thought to play a role in mediating insulin sensitivity, with a relatively low content of saturated fatty acids correlating with higher insulin sensitivity (51). In support of this prior evidence, the present study demonstrated that the change in the sum of all saturated fatty acids within skeletal muscle phospholipids correlated with the change in postprandial insulinemia. Single-leg exercise training has been used to show increased polyunsaturated fatty acid content of skeletal muscle phospholipids in an exercised versus non-exercised leg (52). Since that change was independent of dietary intake, the reduction in the saturated fatty content of phospholipids was likely due to a preferential upregulation of saturated fatty acid oxidation as a result of the higher energy expenditure (53, 54). However, because this previous work involved changes in energy expenditure across experimental conditions, the role of lipid utilization independent of energy expenditure on phospholipid remodeling could not be explored. It should be acknowledged that at the level of individual fatty acids, the only substantial change in the current study was for an increase in the saturated fatty acid stearate with exercise training performed before versus after carbohydrate intake. The present study may, however, lack the statistical power to detect changes in other fatty acids as the skeletal muscle phospholipid analysis was only possible on a limited number of participants where tissue sample size allowed. Therefore, more work is required to characterize the specific fatty acid compositional changes in skeletal muscle phospholipids (and other lipid pools) with changes in nutrient-exercise timing.

AMPK is also nutrient sensitive and contributes to regulation of fatty acid utilization (55), mitochondrial biogenesis (56), and the expression of proteins involved in skeletal muscle glucose uptake, including GLUT4 and AS160 (57–59), which are key players in whole-body insulin sensitivity (60). We observed greater increases in the protein content of AMPK in skeletal muscle with exercise training before versus after nutrient intake. The increase in the GLUT4 content of skeletal muscle we observed with exercise before nutrient provision may be explained by this heightened AMPK response and, in turn, may have contributed to increases in the OGIS index following exercise training before versus after nutrient provision (61). Skeletal muscle AMPK can be activated by increased fatty acid availability, independent of muscle glycogen and AMP concentrations (62). Muscle glycogen utilization can modulate AMPK and GLUT4 mRNA expression with different exercise models (60). However, since we observed no difference in muscle glycogen utilization with altered carbohydrate availability during exercise in the Acute Study, the change in the GLUT4 content with exercise training before versus after carbohydrate ingestion is likely to be attributable to repeated increases in fatty acid availability, potentially through increases in the skeletal muscle AMPK content. However, it should also be noted that the acute and training experiments in this study provided different nutrients as the breakfast (ie, a mixed-macronutrient carbohydrate-rich meal versus a carbohydrate drink). As such, some inferences regarding the translation of the acute responses to the longer-term training responses should be interpreted cautiously. Finally, the AMPK antibody we used detects both isoforms of the catalytic subunits of AMPK (AMPKα1 and α2). In human skeletal muscle, three different complexes have been described [α2β2γ1, α2β2γ3, and α1β2γ1; (63)], and our antibody captured all complexes. Accordingly, we cannot speculate whether a specific heterotrimeric AMPK complex is predominately contributing to the increase in AMPK content we report. As such, the effect of nutrient-exercise timing on AMPK activation warrants further investigation.

The correct targeting and sequestration of GLUT4 into its intracellular insulin-responsive compartments is also important for insulin sensitivity in skeletal muscle (64, 65). Clathrin heavy-chain isoform 22 (CHC22) plays a specialized role in regulating GLUT4 sequestration in human skeletal muscle (66), protecting GLUT4 from degradation (67) and making it more available for insulin-stimulated release. We showed an increase in CHC22 protein levels in exercised muscle (relative to the exercise effects on CHC17 protein levels) with exercise before versus after nutrient provision. As the cognate clathrin CHC17 plays a widespread membrane traffic role in many tissues, CHC17 levels provide a benchmark for general membrane traffic changes compared to those in the GLUT4 pathway (68). The relative increase in CHC22 levels we observed suggests that exercise before nutrient provision not only augments GLUT4 protein levels, but potentially also the machinery necessary for the appropriate sequestration and targeting of GLUT4 to its insulin-responsive compartment. This may lead to improved GLUT4 translocation and contribute to the increases in the OGIS index we observed with exercise training before versus after carbohydrate intake. Further work is now needed to investigate nutrient-exercise interactions and their effect on CHC22 levels due to the small sample size and variability in the CHC22 responses in this study. In addition, the remodeling of skeletal muscle phospholipids could have contributed to the ability of GLUT4 to fuse to the muscle-plasma membrane via less rigid arrays of phospholipid molecules in plasma membranes (69).

The greater increase in AMPK content we observed with exercise before carbohydrate provision did not further augment measured markers of mitochondrial biogenesis in skeletal muscle in response to exercise training in men who are overweight. This is in contrast to prior work demonstrating that carbohydrate ingestion before and during exercise suppresses exercise-induced increases in the content of proteins in skeletal muscle involved in fatty acid transport and oxidation (25). This further highlights that the model of nutrient-exercise timing that we employed (carbohydrate consumption before versus after exercise) might be distinct from other types of nutrient timing. Although changes in skeletal muscle mitochondrial content and/or oxidative capacity may be involved in regulating insulin sensitivity (70), the lack of differential response with exercise before versus after nutrition provision in this study suggests that these factors are unlikely to explain the changes in the OGIS index with the current model of nutrient-exercise timing employed (ie, exercise before versus after breakfast). It is also interesting that the intramuscular adaptations and changes in the OGIS index that we observed occurred in the presence of similar changes in body composition, self-reported daily dietary intake, and total daily energy expenditure with altered nutrient-exercise timing. Notwithstanding other factors that may have contributed to the increases in OGIS with exercise before versus after nutrient ingestion, this highlights lipid metabolism as a potentially important mechanism explaining the improvement in OGIS with regular exercise performed before versus after breakfast.

It should also be noted that the responses observed for OGIS were an interaction between groups, and thus the response to exercise before nutrient provision is an increase relative to the non-exercise control group and the exercise-after-nutrient-intake group. Accordingly, these data may be specific to high-carbohydrate provision, and although this is typical of breakfasts in developed countries, it remains to be seen whether lower-carbohydrate meals produce similar effects. Potential limitations in our work also include the absence of a non-exercise fasting group, which would have allowed us to explore the role of extended morning fasting in the Training Study. However, our prior work has already shown that extended morning fasting in an absence of exercise may impair insulin sensitivity and increase postprandial insulinemia in humans with obesity (71).

To summarize, the present data are the first to show that exercise training before versus after carbohydrate (ie, breakfast) consumption affects responsiveness to moderate-intensity exercise training in men classified as overweight or obese, including greater remodeling of skeletal muscle phospholipids, adaptations of proteins involved in nutrient sensing and glucose transport in skeletal muscle, and increases in and index of OGIS. These data suggest that exercising in a fasted state can augment the adaptive response to exercise, without the need to increase the volume, intensity, or perception of effort of exercise. These responses may be linked to the acute increases in lipid utilization during every bout of exercise performed in the fasted versus the fed state (a difference that is sustained throughout a period of training over 6 weeks). These findings, therefore, have implications for future research and clinical practice. For example, exercise training studies should account for nutrient-exercise timing if aspects of metabolic control are an outcome measure. Secondly, to increase lipid utilization and OGIS with training, endurance-type exercise should be performed before versus after nutrient intake (ie, in the fasted state).

## Abbreviations

ANOV: analysis of variance
AMPK: activated protein kinase
AUC: area under the curve
CHC: clathrin heavy-chain
CHO-EX: carbohydrate-only breakfast before exercise
CON: no-exercise control group
CV: coefficient of variation
EX-CHO: exercise before breakfast carboyhydrate only
IMTG: intramuscular triglyceride
mRNA: messenger RNA
NEFA: non-esterified fatty acid
OGIS: oral glucose insulin sensitivity
OGTT: oral glucose tolerance test
PAEE: physical activity energy expenditure
PAL: physical activity level
RMR: resting metabolic rate
T2D: type 2 diabetes
VO2peak: peak oxygen rate.

## Additional Information

***Disclosure Summary:*** The authors have nothing to disclose.

***Data Availability:*** Raw data and supplemental methods and tables are available as online supporting information (https://researchportal.bath.ac.uk/en/datasets/;https://doi.org/10.15125/BATH-00672).
